# Unusual Neurological Manifestation of Proton Pump Inhibitor: A Case Report of Acute Disseminated Encephalomyelitis and Severe Hyponatremia After Brief Use of Proton Pump Inhibitor

**DOI:** 10.7759/cureus.15571

**Published:** 2021-06-10

**Authors:** Ahmad S Qureshi, Mohammad A Quadri, Babar Javed

**Affiliations:** 1 Critical Care Medicine, National Guard Health Affairs, Al Madinah, SAU

**Keywords:** acute disseminated encephalomyelitis, omeprazole, hyponatremia, mri, female

## Abstract

Hyponatremia is commonly reported after the use of proton pump inhibitors (PPI). While omeprazole is most likely to cause hyponatremia, almost all the PPIs have been reported to cause hyponatremia. The underlying mechanism of PPI-induced hyponatremia is a syndrome of inappropriate antidiuretic hormone (SIADH) secretion which leads to hyponatremia. The hyponatremia can develop with only a few days of exposure to PPI. We present a case of a 56-year-old previously healthy female who was prescribed omeprazole for trivial acid reflux symptoms when she presented to the emergency room for evaluation of generalized weakness. She was discharged home from the emergency room after clinical evaluation as she had essentially normal lab work including a negative COVID PCR test. She subsequently developed progressive weakness of extremities and slurred speech over the next three days. She returned back to the emergency room and was found to have profound hyponatremia with MRI evidence of acute disseminated encephalomyelitis (ADEM). She was treated with hypertonic saline to correct hyponatremia and omeprazole was discontinued. The patient also received pulse dose steroids with improvement in her symptoms.

## Introduction

Drug-induced hyponatremia has been described with a number of medications including non-steroidal anti-inflammatory drugs (NSAIDS), antipsychotics, selective serotonin reuptake inhibitors (SSRIs), and proton pump inhibitors (PPI) [1]. However, there are very few case reports of acute disseminated encephalomyelitis (ADEM) associated with drug-induced hyponatremia [2,3]. ADEM is acute inflammatory neurological disorders of several etiologies. Brighton collaboration has established case definitions for ADEM [4]. ADEM is diagnosed based on clinical and radiological abnormalities which are best seen on MRI imaging of the brain, brainstem, and spinal cord. MRI findings are best seen on T2 weighted images, fluid-attenuated inversion recovery (FLAIR) sequence, and Gadolinium contrast-enhanced images. MRI findings that raise suspicion for ADEM include deep white matter and subcortical lesion of variable size, location and symmetry involving the brain, brainstem and spinal cord. These disorders typically occur one to two weeks after bacterial, viral infections, or vaccination. In some patients, the presentation may be delayed up to three months [5]. Vaccine-induced ADEM has been associated with influenza, human papilloma virus (HPV), pneumococcal conjugate vaccine (PCV), rabies, diphtheria-tetanus-polio, smallpox, measles, mumps, rubella, Japanese B encephalitis, pertussis, and hepatitis B [6,7]. Pallegrino et al. reported that Flu and HPV were most commonly associated with ADEM and together accounted for one-third of cases [7,8]. We now report a case of ADEM related to the use of PPI Omeprazole that developed briskly and led to neurological deficits.

## Case presentation

A 56-year-old previously healthy female presented to the emergency room for evaluation for generalized weakness, body aches and pains as well as trivial acid reflux. Evaluation in the emergency room showed normal clinical examination and lab work including a negative COVID (PCR) test. She was discharged home on symptomatic management. She was prescribed omeprazole and acetaminophen as needed for intermittent acid reflux, body aches, and pain. Over the next four days, the patient developed progressive weakness of the lower extremities with slurred speech to the point that she needed assistance to ambulate. The patient reported poor appetite during this time. She denied polydipsia or excessive water intake. She did not report any impaired sensations, loss of bladder, or bowel control. These symptoms prompted her to return to the emergency room on the fifth day of her initial visit to the emergency department. The patient was alert, awake, and oriented. Vitals signs in the emergency room showed elevated initial blood pressure at 190/95 mm Hg (improved to 156/74 mm Hg without any intervention), a pulse of 95 beats per minute, temperature 37 °C, oxygen saturation of 94% on room air. Her BMI was 28.3 kg/m^2^. Her Glasgow Coma Scale (GCS) was normal at 15/15. Physical examination was remarkable for a normal cardiac, respiratory, and abdominal examinations. Neurologically, there was evidence of 4/5 weakness in bilateral upper extremities and 2/5 weakness in bilateral lower extremities. There was generalized hyporeflexia. There was no sensory impairment. Gait could not be tested. Pertinent laboratory test results are outlined in Table 1.

**Table 1:** Pertinent laboratory test results

Laboratory test	Normal values	Initial emergency room presentation (day 1)	Subsequent emergency room presentation and hospital admission on (day 5)	Comments
Serum sodium	136–145 mmol/L	137	116	Abnormal (low)
Serum uric acid	149–349 umol/L		140	
Random cortisol	102–535 nmol/L		518	
Serum TSH	0.35 ~ 4.94 mIU/L		3.81	
Free T4	9 ~ 19 pmol/L		15.03	
Serum osmolality	276–294 mOsm/kg		236	Abnormal (low)
Urine osmolality	301 ~ 899 mOsm/kg		700	
Urinary sodium	mmol/L		64	
Urinary potassium	mmol/L		36	
Urinary chloride	mmol/L		89	
CSF appearance	Clear		Clear	
CSF protein	0.15–0.4 g/L		1.76	Abnormal (elevated)
CSF glucose	2.21–3.89 mmol/L		4.62	Abnormal (elevated)
CSF WBC	0 ~ 5 × 10 6 /L		1	
CSF RBC	0 ~ 10 × 10 6/L		7	
CSF segments	0-6 %		20%	Abnormal (elevated)
CSF monocytes	15 ~ 45%		64%	Abnormal (elevated)
CSF lymphocytes	40 ~ 80 %		16%	Abnormal (low)
COVID PCR			Negative	

**Figure 1:**
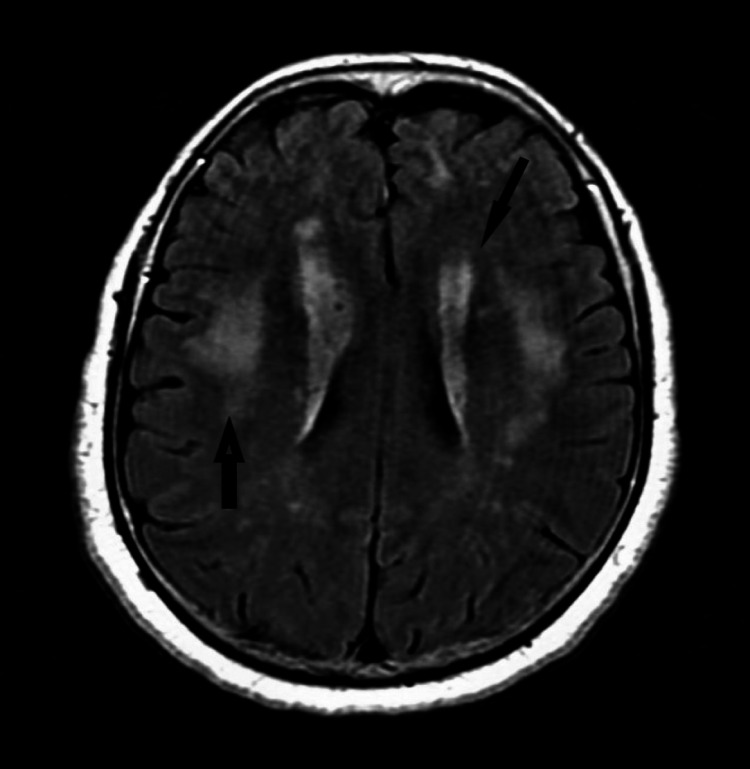
MRI Brain showing patchy areas of asymmetric subcortical lesion on FLAIR sequence images (black arrows)

Chest X-ray was unremarkable. MRI brain showed patchy areas of asymmetric subcortical lesions on FLAIR sequence images which did not show enhancement on contrast-enhanced T1 images with gadolinium (Figure 1). MRI of cervical, thoracic and lumbar spine were normal.

The patient was treated with hypertonic saline 2% with very slow correction of serum sodium over the next 24 hours at the rate of less than 0.5 mEq/hour. Subsequently, serum sodium improved to 133 mEq/L over one week. Serum uric acid also corrected to normal within 48 hours of admission. The patient had taken Omeprazole for four days prior to being admitted to the hospital and was discontinued on admission. The patient received pulse dose steroids using 1 g of methylprednisolone for five days along with physical and occupational therapy. This led to partial improvement of her weakness in lower extremities graded at 3/5 at the time of discharge. The patient did not receive plasmapheresis or intravenous immune globulins (IVIG) during the course of the hospital stay.

## Discussion

To the best of our knowledge, this is the first case report of omeprazole-induced hyponatremia presenting as ADEM. ADEM is multifactorial in etiology and the possibility of this being related to co-existing viral syndrome cannot be entirely ruled out. The patient did not take acetaminophen prescribed to her for symptomatic management of aches and pains. Hyponatremia associated with PPI use especially with omeprazole tends to occur with recent use and typically in elderly female patients. While there is no one MRI brain pattern that is diagnostic of ADEM, it is a combination of clinical suspicion and imaging that is helpful in establishing the diagnosis. ADEM is immune-mediated while MS is genetic in nature. ADEM affects children more often and has an acute onset and relatively good prognosis. On the other hand, multiple sclerosis (MS) tends to affect young adults with gradual onset but when it presents in children it can lead to significant disability over time. A follow-up MRI may be needed to distinguish it from MS as ADEM is, in general, a unimodal entity although in children a multimodal pattern like multiple sclerosis has been described. Mori et al. [2] have described a case of ADEM associated with hyponatremia related to the cerebral disease. However, ADEM is a rare complication associated with medication-induced hyponatremia [3]. Neurological manifestations such as dementia from long-term use of PPIs are well known which is attributed to amyloid protein deposition in neurons and vitamin B-12 deficiency [8,9]. Overall, the great majority of patients with ADEM show significant improvement in neurological deficits over a period of one to six months [10]. While correction of hyponatremia is imperative, for ADEM pulse dose steroids followed by prednisolone taper over subsequent four to six weeks is often needed. Other options for non-responders include IVIG and plasmapheresis [11-13].

## Conclusions

Over the past several decades, PPI has become the preferred treatment for erosive esophagitis, peptic ulcer disease, and *Helicobacter pylori* infections. However, because of the easy availability of generic formulations over the counter, public access to this class of medication has also increased. While PPIs are potent acid suppressants, they are not free from serious side effects like electrolyte imbalances, risk for osteoporosis, and predisposition to Clostridium difficile infection, to name a few. This case report highlights the importance of recognizing the potential of PPIs to cause serious side effects like hyponatremia which can occur even with brief exposure to these medications. It also mandates that physicians recognize that patients who are taking PPIs may present with unusual or unexplained neurological findings, which may be related to the use of this class of medication. Additionally, it also highlights the importance of judicious use of such medications with close clinical follow-up, especially, in elderly females.
